# Draft Genome Sequence of *Bacillus licheniformis* Strain GB2, a Hydrocarbon-Degrading and Plant Growth-Promoting Soil Bacterium

**DOI:** 10.1128/genomeA.00608-16

**Published:** 2016-06-23

**Authors:** Panagiotis Gkorezis, Jonathan Van Hamme, Eric Bottos, Sofie Thijs, Maria Balseiro-Romero, Carmela Monterroso, Petra Suzan Kidd, Francois Rineau, Nele Weyens, Wouter Sillen, Jaco Vangronsveld

**Affiliations:** a Hasselt University, Centre for Environmental Sciences, Diepenbeek, Belgium; b Thompson Rivers University, Department of Biological Sciences, Kamloops, Canada; c University of Santiago de Compostela, Department of Soil Science and Agricultural Chemistry, Santiago de Compostela, Spain; d Instituto de Investigaciones Agrobiológicas de Galicia (IIAG), Consejo Superior de Investigaciones Científicas (CSIC), Santiago de Compostela, Spain

## Abstract

We report the 4.39 Mb draft genome of *Bacillus licheniformis* GB2, a hydrocarbonoclastic Gram-positive bacterium of the family *Bacillaceae*, isolated from diesel-contaminated soil at the Ford Motor Company site in Genk, Belgium. Strain GB2 is an effective plant-growth promoter useful for diesel fuel remediation applications based on plant-bacterium associations.

## GENOME ANNOUNCEMENT

Members of the genus *Bacillus*, and more specifically various species of *Bacillus licheniformis*, have been associated with diesel oil degradation in both pure and mixed bacterial cultures (1). It has been reported that *Bacillus licheniformis* strains can directly and indirectly enhance the growth of plant species such as *Arachis hypogea* (2), *Brassica campestris* (3), and *Vitis vinifera* cv. Malbec (4).

Using gas chromatography (GC) (model 450, Agilent Technologies) coupled to mass spectrometry (MS) (model 220, Agilent Technologies), *B. licheniformis* GB2 was found to degrade up to 41% of diesel range organics over 14 days. Under greenhouse conditions *B. licheniformis* GB2 promoted the growth of hybrid poplar cuttings (*Populus deltoides* × [*trichocarpa* × *deltoides*] cv. Grimminge), as well as *Cytisus striatus* and *Lupinus luteus*, indicating that this bacterial strain has a broad plant host specificity.

For sequencing, an Ion Torrent PGM (Life Technologies, Inc., Carlsbad, CA) was used after extracting DNA from stationary-phase cells of GB2 using techniques as described by Thijs et al. (5).

In total, 718,104 reads (mean length 241 bases) generated 174 Mb of data (>145 M Q20 bases) in Torrent Suite 4.2.1. These were assembled using SPAdes 3.1.0 (6, 7) (uniform coverage mode; k-mers 21, 33, 55, 77, 99) into 24 contigs greater than 500 bp, giving a consensus length of 4,392,282 bp at 20.0× coverage (largest contig 1,288,409 bp; *N*_50_ = 507,764 bp). To identify closely related organisms with complete genomes in NCBI RefSeq, the Rapid Annotations Subsystems Technology (RAST) server (8) was used.

*Bacillus licheniformis* ATCC 14580 (GenBank accession no. CP000002) was used as a reference to reorder the GB2 contigs in Mauve (9). Open reading frame (ORF) prediction and gene annotation was completed out using the PGAP (NCBI) pipeline (10). The genome of *Bacillus licheniformis* GB2 consists of a single circular chromosome (46.1 % G+C content), including 4,586 coding genes that were arranged into pathways using Pathway Tools (11, 12), 420 pseudogenes, 22 rRNAs (5S, 16S, 23S), 77 tRNAs, and 1 noncoding RNA (ncRNA).

Homologues of genes related to plant growth-promoting traits such as nitrogen fixation, inorganic phosphorus solubilization, auxin biosynthesis, as well as acetoin and siderophore production were found, along with copies of genes for alkane utilization including alkane monooxygenase (*alkB*), and aldehyde dehydrogenase (*alkH*). The lower naphthalene pathway is partially present in GB2.

The use of *B. licheniformis* GB2 as a plant growth-promoting inoculant for enhancing remediation of diesel-contaminated sites based on plant-bacteria partnerships is being investigated.

### Nucleotide sequence accession numbers.

This whole-genome shotgun project has been deposited at DDBJ/EMBL/GenBank under the accession number JYGX00000000. The version described in this paper is version JYGX01000000.

## References

[1] Purwanti IF, Abdullah SRS, Hamzah A, Idris M, Basri H, Mukhlisin M, Latif MT 2015 Biodegradation of diesel by bacteria isolated from *Scirpus mucronatus* rhizosphere in diesel-contaminated sand. Adv Sci Lett 21:140–143. doi:10.1166/asl.2015.5843.

[2] Goswami D, Dhandhukia P, Patel P, Thakker JN 2014 Screening of PGPR from saline desert of Kutch: growth promotion in *Arachis hypogea* by *Bacillus licheniformis* A2. Microbiol Res 169:66–75. doi:10.1016/j.micres.2013.07.004. 23896166

[3] Maheshwari DK, Dubey RC, Agarwal M, Dheeman S, Aeron A, Bajpai VK 2015 Carrier based formulations of biocoenotic consortia of disease suppressive *Pseudomonas aeruginosa* KRP1 and *Bacillus licheniformis* KRB1. Ecol Eng 81:272–277. doi:10.1016/j.ecoleng.2015.04.066.

[4] Salomon MV, Bottini R, de Souza Filho GA, Cohen AC, Moreno D, Gil M, Piccoli P 2014 Bacteria isolated from roots and rhizosphere of *Vitis vinifera* retard water losses, induce abscisic acid accumulation and synthesis of defense-related terpenes in *in vitro* cultured grapevine. Physiol Plant 151:359–374. doi:10.1111/ppl.12117. 24118032

[5] Thijs S, Van Hamme J, Gkorezis P, Rineau F, Weyens N, Vangronsveld J 2014 Draft genome sequence of *Raoultella ornithinolytica* TNT, a trinitrotoluene-denitrating and plant growth-promoting strain isolated from explosive-contaminated soil. Genome Announc 2(3):e00491-14. doi:10.1128/genomeA.00491-14. 24874678 PMC4038883

[6] Bankevich A, Nurk S, Antipov D, Gurevich AA, Dvorkin M, Kulikov AS, Lesin VM, Nikolenko SI, Pham S, Prjibelski AD, Pyshkin AV, Sirotkin AV, Vyahhi N, Tesler G, Alekseyev MA, Pevzner PA 2012 SPAdes: a new genome assembly algorithm and its applications to single-cell sequencing. J Comput Biol 19:455–477. doi:10.1089/cmb.2012.0021. 22506599 PMC3342519

[7] Gurevich A, Saveliev V, Vyahhi N, Tesler G 2013 QUAST: quality assessment tool for genome assemblies. Bioinformatics 29:1072–1075. doi:10.1093/bioinformatics/btt086. 23422339 PMC3624806

[8] Aziz RK, Bartels D, Best AA, DeJongh M, Disz T, Edwards RA, Formsma K, Gerdes S, Glass EM, Kubal M, Meyer F, Olsen GJ, Olson R, Osterman AL, Overbeek RA, McNeil LK, Paarmann D, Paczian T, Parrello B, Pusch GD, Reich C, Stevens R, Vassieva O, Vonstein V, Wilke A, Zagnitko O 2008 The RAST server: Rapid Annotations using Subsystems Technology. BMC Genomics 9:75. doi:10.1186/1471-2164-9-75. 18261238 PMC2265698

[9] Rissman AI, Mau B, Biehl BS, Darling AE, Glasner JD, Perna NT 2009 Reordering contigs of draft genomes using the mauve aligner. Bioinformatics 25:2071–2073. doi:10.1093/bioinformatics/btp356. 19515959 PMC2723005

[10] Angiuoli SV, Gussman A, Klimke W, Cochrane G, Field D, Garrity G, Kodira CD, Kyrpides N, Madupu R, Markowitz V, Tatusova T, Thomson N, White O 2008 Toward an online repository of standard operating procedures (SOPs) for (meta) genomic annotation. Omics J Integr Biol 12:137–141. doi:10.1089/omi.2008.0017. PMC3196215 18416670

[11] Caspi R, Altman T, Billington R, Dreher K, Foerster H, Fulcher CA, Holland TA, Keseler IM, Kothari A, Kubo A, Krummenacker M, Latendresse M, Mueller LA, Ong Q, Paley S, Subhraveti P, Weaver DS, Weerasinghe D, Zhang P, Karp PD 2014 The MetaCyc database of metabolic pathways and enzymes and the BioCyc collection of pathway/genome databases. Nucleic Acids Res 42:D459–D471. doi:10.1093/nar/gkt1103. 24225315 PMC3964957

[12] Karp PD, Paley S, Romero P 2002 The pathway tools software. Bioinformatics 18:S225–S232. doi:10.1093/bioinformatics/18.suppl_1.S225. 12169551

